# Knowledge and valuation of Andean agroforestry species: the role of sex, age, and migration among members of a rural community in Bolivia

**DOI:** 10.1186/1746-4269-9-83

**Published:** 2013-12-20

**Authors:** Regine Brandt, Sarah-Lan Mathez-Stiefel, Susanne Lachmuth, Isabell Hensen, Stephan Rist

**Affiliations:** 1 Institute of Biology/Geobotany and Botanical Garden, Martin-Luther-University, Am Kirchtor 1, 06108 Halle/Saale, Germany; 2 Centre for Development and Environment (CDE), University of Bern, Hallerstrasse 10, 3012 Bern, Switzerland; 3 German Centre for Integrative Biodiversity Research (iDiv) Halle-Jena-Leipzig, Deutscher Platz 5e, 04103 Leipzig, Germany

**Keywords:** Cultural importance, Intracultural variation, Plant use knowledge, Quantitative ethnobotany, Woody species

## Abstract

**Background:**

Agroforestry is a sustainable land use method with a long tradition in the Bolivian Andes. A better understanding of people’s knowledge and valuation of woody species can help to adjust actor-oriented agroforestry systems. In this case study, carried out in a peasant community of the Bolivian Andes, we aimed at calculating the cultural importance of selected agroforestry species, and at analysing the intracultural variation in the cultural importance and knowledge of plants according to peasants’ sex, age, and migration.

**Methods:**

Data collection was based on semi-structured interviews and freelisting exercises. Two ethnobotanical indices (Composite Salience, Cultural Importance) were used for calculating the cultural importance of plants. Intracultural variation in the cultural importance and knowledge of plants was detected by using linear and generalised linear (mixed) models.

**Results and discussion:**

The culturally most important woody species were mainly trees and exotic species (e.g. *Schinus molle*, *Prosopis laevigata*, *Eucalyptus globulus*). We found that knowledge and valuation of plants increased with age but that they were lower for migrants; sex, by contrast, played a minor role. The age effects possibly result from decreasing ecological apparency of valuable native species, and their substitution by exotic marketable trees, loss of traditional plant uses or the use of other materials (e.g. plastic) instead of wood. Decreasing dedication to traditional farming may have led to successive abandonment of traditional tool uses, and the overall transformation of woody plant use is possibly related to diminishing medicinal knowledge.

**Conclusions:**

Age and migration affect how people value woody species and what they know about their uses. For this reason, we recommend paying particular attention to the potential of native species, which could open promising perspectives especially for the young migrating peasant generation and draw their interest in agroforestry. These native species should be ecologically sound and selected on their potential to provide subsistence and promising commercial uses. In addition to offering socio-economic and environmental services, agroforestry initiatives using native trees and shrubs can play a crucial role in recovering elements of the lost ancient landscape that still forms part of local people’s collective identity.

## Abstract (es)

**Abstract:**

**Introducción**

Agroforestería es un método de uso de la tierra sostenible con una larga tradición en los Andes bolivianos. Un mejor entendimiento del conocimiento y valoración de las especies leñosas puede apoyar la mejora de los sistemas agroforestales orientados a actores locales. Este estudio de caso se realizó en una comunidad campesina de los Andes bolivianos con los objetivos de calcular la importancia cultural de especies agroforestales seleccionadas y analizar la variación intracultural del conocimiento sobre las mismas y de su valoración según sexo, edad y migración de los campesinos.

La colecta de datos se basó en entrevistas semiestructuradas y listados libres. Se usaron dos índices etnobotánicos (Composite Salience, Cultural Importance) para calcular la importancia cultural de plantas. La variación intracultural de la importancia de las plantas y del conocimiento sobre las mismas se evaluó mediante modelos lineares y modelos lineares generalizados (mixtos).

**Resultados y discusión**

Las especies leñosas culturalmente más importantes fueron principalmente árboles y especies exóticas (p.ej. *Schinus molle*, *Prosopis laevigata*, *Eucalyptus globulus*). El sexo desempeñó un menor rol al contrario a los efectos positivos de la edad y efectos negativos de la migración sobre la importancia cultural de las plantas y el conocimiento sobre las mismas. El efecto de la edad posiblemente resultó debido a la disminución de apariencia ecológica de especies nativas y su reemplazo por árboles exóticos, la pérdida de usos tradicionales o la utilización de otros materiales (p.ej. plástico) en vez de madera. La menor dedicación a la agricultura tradicional podía haber implicado un abandono sucesivo de herramientas tradicionales, y una transformación general del uso de las plantas leñosas posiblemente se relacione con la disminución del conocimiento medicinal.

**Conclusiones**

Debido a los efectos de edad y migración sobre la valoración de plantas leñosas y el conocimiento sobre los usos de las mismas, recomendamos que se debe prestar una atención particular al potencial de especies nativas para abrir perspectivas prometedoras a la generación campesina joven migrante y aumentar su interés en la agroforestería. Por eso, se deberían seleccionar especies ecológicamente compatibles según su potencial para proporcionar la subsistencia y posibles usos comerciales. Aparte, las iniciativas agroforestales usando árboles y arbustos nativos pueden jugar un rol importante en la recuperación del paisaje tradicional que todavía forma parte de la identidad colectiva de los campesinos locales.

## Background

Measuring the cultural importance of plants is a key issue in quantitative ethnobotanical studies [1,2] and a valuable tool for sustainable land use practices such as agroforestry [3,4]. Ethnobotany provides numerous methods for obtaining quantifiable data to identify cultural plant values within their given socioecological context [5]. For example, “freelisting” enables rapid data sampling based on the assumption that the respondents mention culturally important species more frequently and earlier than others [6,7]. Whether people perceive plants as culturally important or not depends on their “ecological apparency”. This hypothesis, adapted to ethnobotanical studies by Phillips and Gentry [8], proposes that the most common and accessible species are those which are more used and valued (see also [9]). Additionally, the quality, intensity, and exclusivity of plant uses should be considered [10]. This is commonly explored with in-depth, semi-structured interviews that focus on exhaustive inventories of plant knowledge including its theoretical dimensions (passive knowledge) [11]. In contrast, freelisting does not produce exhaustive inventories, and instead aims at obtaining information on practical uses (active knowledge) [6,11,12]. Combining methods that collect and analyse data on active and passive knowledge provides a broader understanding of the cultural importance of plants and improves the reliability of the results compared to applying single methods. It also provides interesting insights on the processes of knowledge transmission [13,14].

The cultural importance of plants is commonly estimated by considering the level of agreement among the interviewees about the knowledge underlying plant use [11,13], which is termed “informant consensus”, e.g. [15]. This approach, however, carries the risk of overlooking the social distribution of knowledge, which is most often not equally shared within a certain cultural group [11]. Consequently, in the last decade there was growing interest in analysing the factors that cause intracultural variation [12,14]. Mainly reported in previous studies are age and sex [16,17]. In addition, factors often related to acculturation such as migration [18-20], market integration [21], and formal education [17] have also been reported to have complex effects on ethnobotanical knowledge. Analysing these factors and the dynamics behind intracultural variation is fundamental for understanding the processes of transmission, transformation, recovery, or loss of ethnobotanical knowledge [22-25].

**Figure 1:**
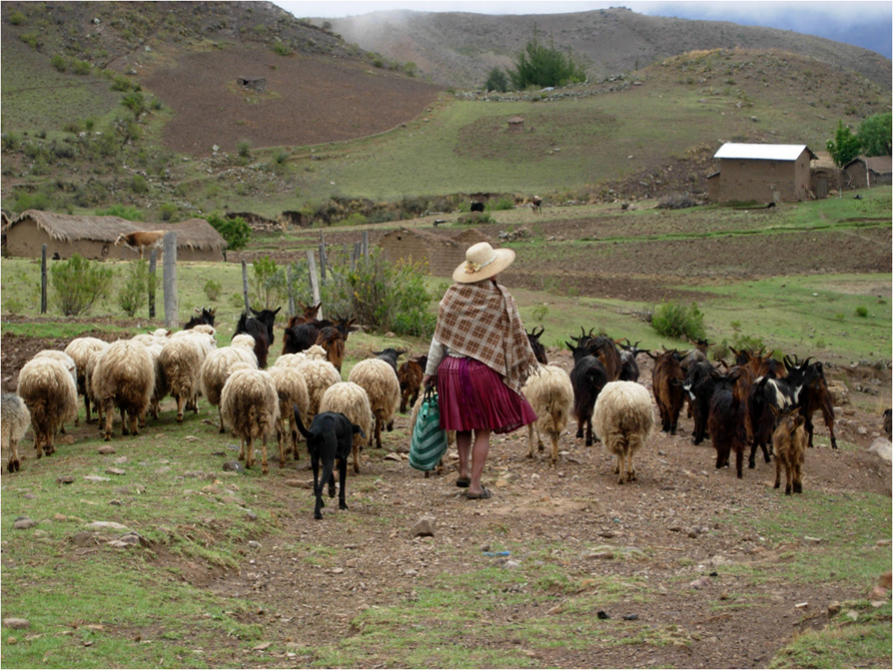
**Livestock rearing.** Photograph by S.-L. Mathez-Stiefel.

**Figure 2:**
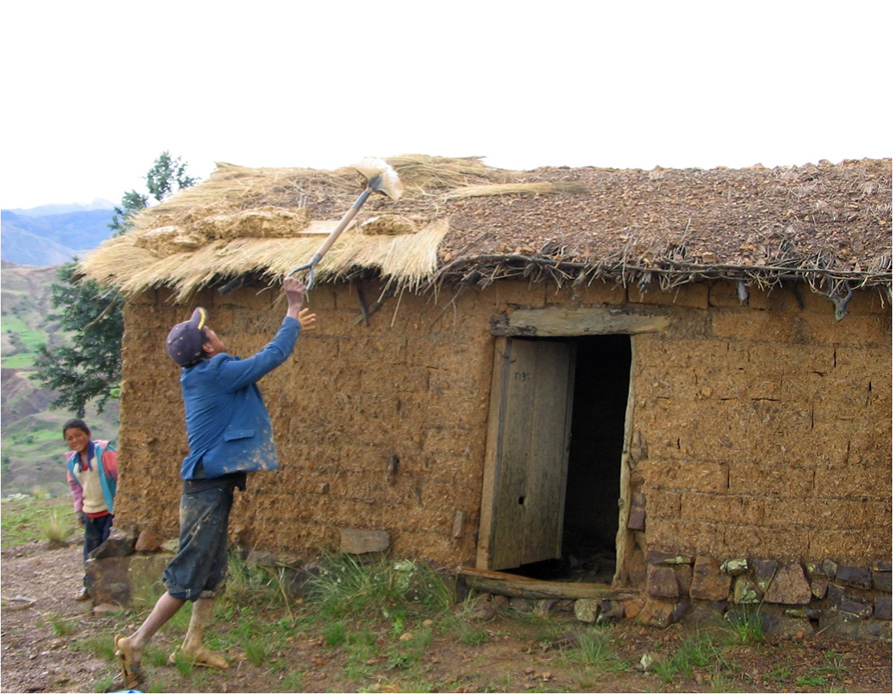
**Reparation of roof cover by using woody branches, straw and loam.** Photograph by R. Brandt.

A better understanding of the intracultural variation in plant knowledge and valuation may help to improve sustainable agroforestry systems [3,26] that aim at providing socio-economic benefits for subsistence and commercial use (e.g. production of fodder, fuel, fruits [4,27]), and environmental services in agro-pastoral landscapes (e.g. soil and biodiversity conservation, carbon sequestration [28-30]). In the Bolivian Andes, agroforestry has a long tradition, dating back to before the arrival of the Spanish in the 1530s. However, agroforestry systems using native woody species (e.g. *Alnus acuminata*, *Buddleja* spp., *Schinus molle*) are rarely actively implemented today compared to plantations of the more productive exotic genus *Eucalyptus*[31], because peasants prefer investing in land use systems that are more profitable in the short term than alternative use systems that are more promising in the long term [32]. To reverse this trend, drawing attention to the cultural importance attributed to plants was shown to be a key factor in motivating peasants to better manage the woody species growing on their farm land [3]. Scientific insights into the intracultural variation in such valuation could help optimise agroforestry management [26]. For instance, sex-specific plant valuation and knowledge should be considered in the Bolivian Andes, where both women and men play crucial but different and complementary roles in agro-pastoral production and livelihoods. Among women’s labour domains are domestic work, child care, livestock rearing (Figure 1), seed conservation, and fuelwood collection, while men are more involved in soil management, construction of buildings (Figure 2), manufacturing of tools, and temporary off-farm labour [33]. Such gender roles define the social groups in which people participate and communicate, and thus experience, learn, and share knowledge [34]. This includes knowledge and cultural importance of plants and their uses [10]. Moreover, age-specific variability should be considered, because the cultural importance of plants and its underlying knowledge are dynamic values. They transform over time and thus may vary from one generation to the next due to abrupt or gradual changes in how people use or do not use plants in changing living conditions [10,35]. In the Bolivian highlands and lowlands, rural out-migration to urban centres strongly influences peasants’ livelihoods. Young adults in particular migrate to seek better income and access to basic services such as healthcare, education, and infrastructure [36]. These processes can increase the generational differences in plant-related knowledge and valuation, and can also cause loss of traditional knowledge among the youth [37,38].

Our study aimed at investigating the cultural importance and knowledge of woody species with potential for use in agroforestry in a rural community of the Bolivian Andes, by analysing their intracultural variation. Particularly, we wanted to test the hypotheses that (1) there are sex-specific differences in ethnobotanical knowledge and valuation, in accordance with existing gender roles, and there is a decrease in knowledge and valuation of plants (2) among the youth and (3) as a consequence of migration. To achieve this, we calculated the cultural importance of 14 selected woody plant species, which were assessed among the most valuable plants for use in agroforestry in the context of the studied community [4], by using two different methods of data collection and analysis respectively, and assessed the intracultural variation in the cultural importance of plants by distinguishing between the community members’ sex, age, and migratory activity. We also evaluated the effects of sex, age, and migration on knowledge of the uses of these 14 woody plants.

## Methods

### Research area

**Figure 3:**
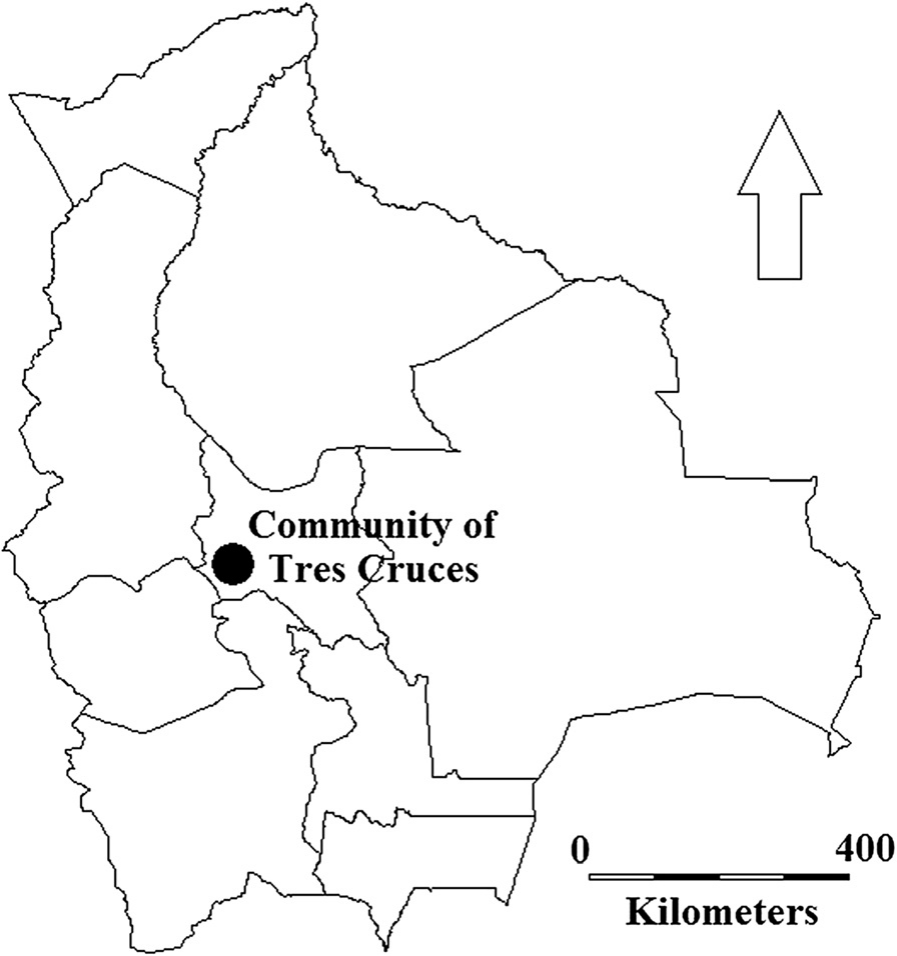
**Research area.** The research was conducted in the indigenous rural community of Tres Cruces (2,760–3,830 m.a.s.l.) in the province of Tapacarí, department of Cochabamba, Bolivia. Map elaborated with DIVA-GIS [40].

**Figure 4:**
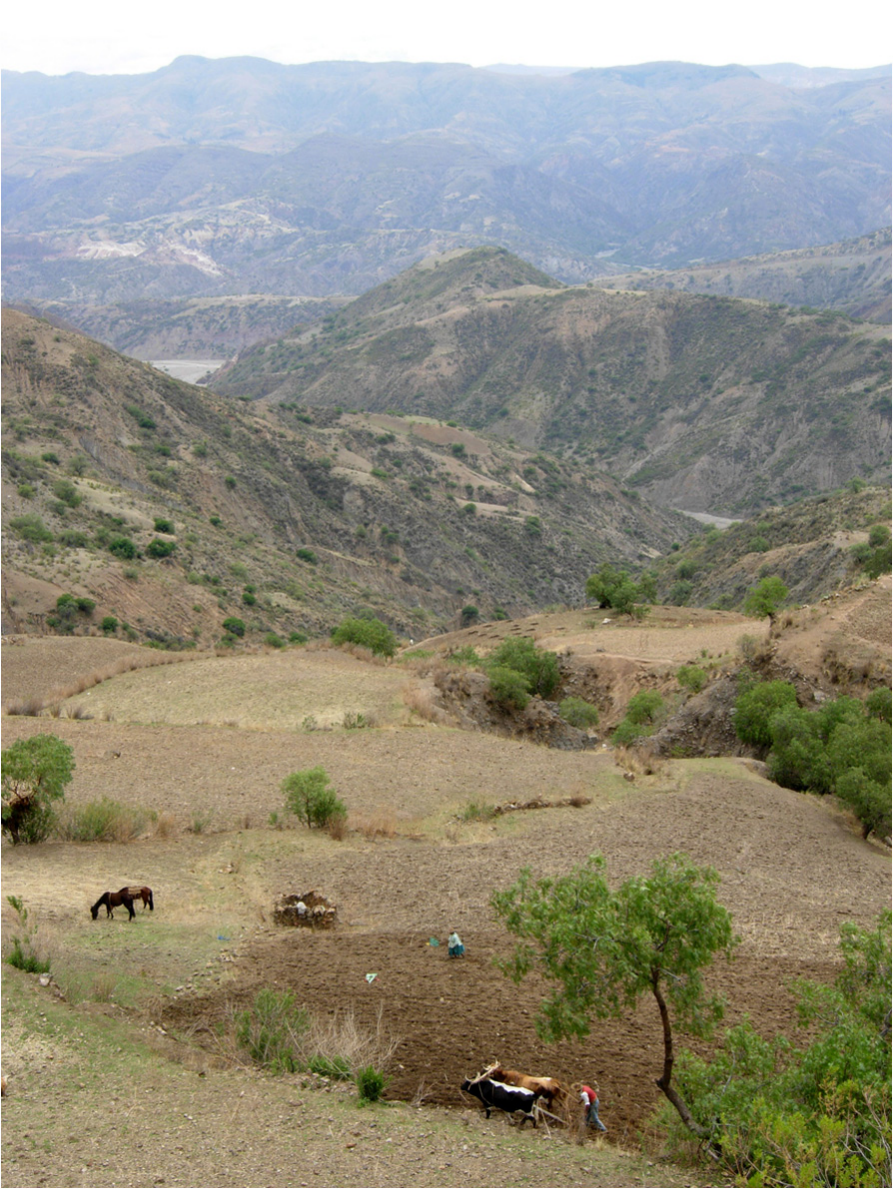
**Landscape of the research area.** Photograph by S.-L. Mathez-Stiefel.

The present study took place in the indigenous rural community Tres Cruces (17°28′–17°30′ S, 66°27′–66°29′ W, ~850 ha, 2,760–3,830 m.a.s.l.) situated in the sub-central Waka Playa of the province of Tapacarí, Cochabamba, Bolivia (Figures 3 and 4). This semi-arid region receives an average of 600 mm of annual precipitation, with >80% of the rainfall occurring between November and March (Ramadas No. 401–17, 1971–2003, Bolivian National Meteorology and Hydrology Service, SENAMHI). Annual mean temperature is about 11°C (Honorable Alcaldía Municipal de Tapacarí, Ajuste del plan de desarrollo municipal Tapacarí 2003–2007). From a biogeographical perspective, the study area extends over the Peruvian Puna Province, in transition to the Bolivian-Tucuman Province, and includes both altitudinal levels puna and prepuna [39]. Natural vegetation of woody species, such as the frequently-growing *Baccharis dracunculifolia* and *Cestrum parqui*, consists of hedges and shrublands on field margins, waysides, stony terrace walls, fallow land, and in ravines [4]. Exotic trees and shrubs (e.g. *Eucalyptus* spp., *Pinus* spp., *Spartium junceum*) were heavily promoted during the course of a participatory rural development project (1999–2002). The population of Tres Cruces consists of 50 indigenous Quechua-speaking families, some inhabitants being bilingual with Spanish. They depend on small-scale subsistence farming with 2 to 6 ha land per household. They cultivate tubers (e.g. *Solanum tuberosum, Ullucus tuberosum*), cereals (e.g. *Zea mays, Triticum sativum, Chenopodium quinoa*), vegetables and fruits, and rear livestock (ovine, caprine, bovine). Farming is complemented by temporary and permanent off-farm activities in the lowlands and urban centres.

### Ethnobotanical data collection

The study was conducted within a development programme for the conservation and enhancement of biocultural diversity in the Bolivian, Peruvian, and Ecuadorian Andes [41]. Following a presentation of the research objectives, prior informed consent was obtained orally from the syndicate assembly of the community of Tres Cruces, the leaders of the sub-central Waka Playa, and from each person interviewed. Data were collected by the main author from January to December 2007 by applying two methods: freelisting [6] on the importance of all local woody species, and semi-structured interviews [11] on the uses of 14 selected local woody species (including trees, shrubs, and sub-shrubs). Due to the research time constraints, we focused our data analysis only on the selected species. They were chosen for their promising potential use in agroforestry, based on their evaluations in an explorative study conducted in the same research area [4] (Table 1). The criteria for plant selection were high, integrated ecological, economic, and sociocultural plant values, high ecological apparency, and absence of negative plant attributes (e.g. toxicity for livestock). Freelisting exercises and semi-structured interviews were conducted with twenty women and twenty men (approximately 25% of the total population). Participants were evenly spread over four age classes: (1) <20 years, (2) 20–39 years, (3) 40–59 years, (4) ≥60 years, with five women and five men per age class. In accordance with Phillips and Gentry [15], participants were interviewed individually in order to avoid cross-influences between the responses. Languages used were Spanish or Quechua, in the latter case with support of an interpreter. Each interview started by identifying whether the participant was a permanent resident of the community, a temporary migrant having a residence both within and outside the community, or a permanent migrant having his/her main residence outside the community. The participants were then asked to freelist their favourite (n = 2-4) and other important local woody species (plant reports) and to explain the reasons for these preferences. The local (vernacular) plant names mentioned were related to their scientific names based on a previous study conducted in the same research area [4], in which collected plant vouchers were identified by comparison with plant material from the herbarium of Cochabamba (BOLV) and in consultation with specialists. In addition, semi-structured interviews were conducted, during which the participants were asked whether and how they used the 14 selected plants for the following nine use-categories (use-reports), based on [4]: (1) construction (house building), (2) environmental use (e.g. soil management), (3) field use (e.g. livestock fence, shelter), (4) fodder, (5) food (including beverages), (6) fuel, (7) medicine (including spiritual healing), (8) tools (e.g. plough, broom), and (9) other use (e.g. commercialisation, domestic uses such as kitchenware and furniture, social and spiritual uses including ornaments and rituals not performed as healing practices). This allowed a collection of exhaustive inventories of use-types per use-category for each species (e.g. participant x mentioned that *Minthostachys ovata,* k’oa muña is used in case of stomach ache and influenza; n = 2 use-types in use-category “medicine”). The interviews were recorded, then translated from Quechua to Spanish and transcribed.

**Table 1:** Characteristics of selected local woody species

**#**	**Species**	**Vernacular name**	**Family**	**Life-form**	**Origin**
1	*Baccharis dracunculifolia* DC.	T’ola	Asteraceae	Shrub	Native
2	*Berberis commutata* Eichler	Churisik’e	Berberidaceae	Shrub	Native
3	*Buddleja coriacea* Remy	Kishuara	Buddlejaceae	Tree	Native (cultivated)
4	*Clinopodium bolivianum* (Benth.) Kuntze	Chini muña, muña	Lamiaceae	Subshrub	Native
5	*Eucalyptus globulus* Labill.	Eucalipto, kalisto	Myrtaceae	Tree	Exotic
6	*Gynoxys psilophylla* Klatt	K’apa towi, loma towi, k’apa k’apa	Asteraceae	Tree	Native
7	*Kaunia saltensis* (Hieron.) R.M. King & H. Rob.	(Jaya) towi	Asteraceae	Shrub	Native
8	*Lepechinia graveolens* (Regel) Epling	Raqacho, raga raga	Lamiaceae	Shrub	Native
9	*Minthostachys ovata* (Briq.) Epling	K‘oa muña, muña	Lamiaceae	Subshrub	Native
10	*Polylepis subtusalbida* (Bitter) M. Kessler & Schmidt-Leb.	Kewiña, queñua	Rosaceae	Tree	Native
11	*Prosopis laevigata* (Humb. & Bonpl. ex Willd.) M.C. Johnst.	Thaqo, algarrobo	Leguminosae	Tree	Native
12	*Sambucus peruviana* Kunth	Sauco	Caprifoliaceae	Tree	Exotic
13	*Schinus molle* L.	Molle	Anacardiaceae	Tree	Native
14	*Senna aymara* H.S. Irwin & Barneby	Motocho, motochila	Leguminosae	Shrub	Native

Selection based on species’ high integrated plant values: ecological (EV), economic (RI), and sociocultural (ICI) plant values, and high ecological apparency (IV) (provided in Brandt et al. [4]).

### Data analysis

The cultural importance of 14 selected woody species (Table 1) and the intracultural variation according to sex (female, male), age (<20 years, 20–39 years, 40–59 years, ≥60 years) and migration (“no_young”: no migration < 40 years, “no_old”: no migration ≥ 40 years, “temp”: temporary migration, “perm”: permanent migration; all temporary and permanent migrants interviewed were < 40 years) were calculated based on two different indices: Composite Salience (Composite S) [6,7] was applied for data obtained by freelisting (active knowledge [6,11,12]), and Cultural Importance (CI) for data gained from semi-structured interviews (passive knowledge) [11]. In order to calculate Composite S, first the salience of each selected plant for each participant was determined by dividing the plant’s inverse rank in the participant’s freelist by the total number of plants mentioned by this participant. Then, Composite S per plant was calculated by summing up the salience scores of each plant and dividing them by the number of participants (n = 40). Thus, the index theoretically ranges between 0 (not mentioned by any participant; not salient) and 1 (mentioned by all participants in first position; highly salient) [6,7]. It was further computed for each actor group per plant and factor (age, sex, and migration; as previously defined). In order to estimate CI, we first grouped all use-reports per plant and participant into the nine use-categories described above (mentioned: 1; not mentioned: 0), and reported the total number of use-types mentioned per use-category, plant, and participant. CI was then calculated for each species and for each actor group (age, sex, and migration; as previously defined) by summing up the proportions of informants that mentioned each use-category. Theoretically, CI may in this case range from 0 (no use-category mentioned by any participant; no cultural importance) to 9 (all use-categories mentioned by all participants; maximal cultural importance) [11].

Statistical analyses were performed in R software for statistical computing and graphics, version 2.14.0 [42]. First, we examined whether Composite S correlated to CI for the 14 selected species by using Spearman rank correlation tests. We applied one-way analyses of variance (ANOVA) using linear models to analyse the effects of the explanatory variables sex, age, and migration (as previously defined) on Composite S and CI (response variables) (see Tables 2 and 3). The two response variables were arcsine square-root transformed to achieve residual normal distribution. Subsequently, Tukey post-hoc tests were applied for pairwise comparisons of means. We also used two-sided binomial tests to compare the probabilities of use-categories being mentioned for each species. They allowed us to check whether previously detected significant intracultural variation in CI was a result of significant differences in the relative importance of one or more specific use-categories between the respective actor groups [43]. Furthermore, analyses of covariance (ANCOVA) were applied using generalised linear models (GLM, R package “stats”, function “glm”, [42]) to evaluate whether the number of use-types mentioned per species varied depending on the participants’ socio-economic characteristics (sex, age, migration). In these analyses, the interviewees’ age was included as a continuous variable. Due to the low number of migrants, temporary and permanent migrants were grouped into one category (yes: migrant; no: resident). All possible two-way interactions among the explanatory variables were included in the maximal models. GLMs are adequate for count data as response variables with Poisson error distribution. If GLMs showed over-dispersion (mean > variance; assessed by calculating residual deviance/residual degrees of freedom) they were re-fitted as quasi-GLMs [43]. Additionally, we analysed the fixed effects of sex, age, migration (as previously defined), and all possible two-way interactions among them on the number of use-types mentioned within use-categories (n = 9). To avoid pseudo-replication, we took into account crossed random effects of species and participants, using generalised linear mixed models (GLMM, R package “lme4”, function “lmer”, [44]). GLMMs are suitable for count data as response variables with Poisson error distribution [45]. All maximal models were simplified in a stepwise-backward procedure based on likelihood ratio tests (chi-square, χ2), or F-tests in case of quasi-GLMs [43]. All non-significant terms (*p* > 0.05) (“ns”, see Tables 4 and 5) were removed to obtain minimal adequate models for each response variable.

**Table 2:** Composite Salience of woody species

**Species**	**Composite Salience**												
	**Total**			**Sex**		**Age**				**Migration**			
	**# part**	**# cit**	**compS**	**female**	**male**	**<20**^**a**^	**20-39**^**b**^	**40-59**^**c**^	≥**60**^**d**^	**no_young**^**a**^	**no_old**^**b**^	**temp**^**c**^	**perm**^**d**^
**BD**	40	33	0.53	0.47 ± 0.31	0.60 ± 0.38	0.47 ± 0.41	0.49 ± 0.27	0.46 ± 0.40	0.72 ± 0.29	0.42 ± 0.30	0.59 ± 0.36	0.41 ± 0.34	0.63 ± 0.38
**BeC**	40	0	0.00	0.00 ± 0.00	0.00 ± 0.00	0.00 ± 0.00	0.00 ± 0.00	0.00 ± 0.00	0.00 ± 0.00	0.00 ± 0.00	0.00 ± 0.00	0.00 ± 0.00	0.00 ± 0.00
**BuC**	40	4	0.03	0.00 ± 0.00	0.05 ± 0.13	0.00 ± 0.00	0.00 ± 0.00	0.07 ± 0.17	0.03 ± 0.08	0.00 ± 0.00	0.05 ± 0.13	0.00 ± 0.00	0.00 ± 0.00
**CB**	40	1	0.02	0.04 ± 0.18	0.00 ± 0.00	0.00 ± 0.00	0.08 ± 0.25	0.00 ± 0.00	0.00 ± 0.00	0.00 ± 0.00	0.00 ± 0.00	0.00 ± 0.00	0.13 ± 0.33
**EG**	40	38	0.71	0.79 ± 0.33	0.63 ± 0.34	0.81 ± 0.29	0.75 ± 0.38	0.69 ± 0.27	0.60 ± 0.40	0.94 ±0.12*^d^	0.64 ± 0.34	0.90 ± 0.24*^d^	0.45 ± 0.38*^a,c^
**GP**	40	1	0.01	0.00 ± 0.00	0.02 ± 0.10	0.00 ± 0.00	0.00 ± 0.00	0.05 ± 0.14	0.00 ± 0.00	0.00 ± 0.00	0.02 ± 0.10	0.00 ± 0.00	0.00 ± 0.00
**KS**	40	6	0.05	0.01 ± 0.04	0.08 ± 0.19	0.00 ± 0.00	0.01 ± 0.03	0.07 ± 0.17	0.11 ± 0.21	0.01 ± 0.03	0.09 ± 0.19	0.00 ± 0.00	0.00 ± 0.00
**LG**	40	18	0.21	0.10 ± 0.16*	0.33 ± 0.36*	0.01 ± 0.05*	0.33 ± 0.33*	0.18 ± 0.29	0.32 ± 0.36	0.09 ± 0.15	0.25 ± 0.32	0.15 ± 0.25	0.30 ± 0.42
**MO**	40	2	0.02	0.05 ± 0.15	0.00 ± 0.00	0.03 ± 0.11	0.06 ± 0.19	0.00 ± 0.00	0.00 ± 0.00	0.04 ± 0.12	0.00 ± 0.00	0.00 ± 0.00	0.10 ± 0.24
**PS**	40	6	0.09	0.07 ± 0.21	0.11 ± 0.26	0.00 ± 0.00*^d^	0.00 ± 0.00*^d^	0.10 ± 0.23	0.25 ± 0.37*^a,b^	0.00 ± 0.00	0.18 ± 0.31	0.00 ± 0.00	0.00 ± 0.00
**PL**	40	28	0.47	0.38 ± 0.39	0.55 ± 0.34	0.19 ± 0.26*^c,d^	0.38 ± 0.32	0.73 ± 0.29*^a^	0.56 ± 0.40*^a^	0.26 ± 0.31*	0.65 ± 0.35*	0.32 ± 0.37	0.29 ± 0.26
**SP**	40	6	0.07	0.07 ± 0.19	0.08 ± 0.24	0.16 ± 0.35	0.08 ± 0.20	0.00 ± 0.00	0.05 ± 0.13	0.28 ± 0.40*	0.03 ± 0.09*	0.00 ± 0.00	0.02 ± 0.05
**SM**	40	33	0.63	0.64 ± 0.37	0.62 ± 0.37	0.55 ± 0.35	0.56 ± 0.38	0.78 ± 0.37	0.63 ± 0.37	0.69 ± 0.23	0.71 ± 0.37	0.63 ± 0.39	0.30 ± 0.37
**SA**	40	19	0.20	0.20 ± 0.25	0.20 ± 0.30	0.10 ± 0.23	0.12 ± 0.18	0.19 ± 0.26	0.38 ± 0.33	0.07 ± 0.13	0.29 ± 0.31	0.08 ± 0.09	0.20 ± 0.32

Composite Salience (compS; arcsine square-root transformed) of 14 selected local woody species in dependence on the participants’ socio-economic background (sex, age, migration). Sex: female (n = 20), male (n = 20). Age: <20 years (n = 10), 20–39 years (n = 10), 40–59 years (n = 10), ≥60 years (n = 10). Migration: no_young (no migration < 40 years, n = 8), no_old (no migration ≥ 40 years, n = 20), temp (temporary migration, n = 6), perm (permanent migration, n = 6). Number of participants (# part). Number of citations (# cit). Data shown: mean values ± standard deviation. Significant differences between groups (a-d) determined by Tukey post-hoc tests. Level of significance: *, *p* < 0.05.

**Table 3:** Cultural Importance of woody species

	**Cultural Importance**													
**Species**	**Total**				**Sex**		**Age**				**Migration**			
	**# part**	**# cit**	**# cat**	**CI**	**female**	**male**	**<20**^**a**^	**20-39**^**b**^	**40-59**^**c**^	≥**60**^**d**^	**no_young**^**a**^	**no_old**^**b**^	**temp**^**c**^	**perm**^**d**^
**BD**	40	193	8	4.83	5.05 ± 1.15	4.60 ± 1.39	4.70 ± 1.57	4.70 ± 1.16	4.80 ± 1.23	5.10 ± 1.29	5.00 ± 1.31	4.95 ± 1.23	3.83 ± 1.60	5.17 ± 0.75
**BeC**	40	122	9	3.05	3.05 ± 1.70	3.05 ± 2.06	2.00 ± 1.63*	2.40 ± 1.35	4.30 ± 1.25*	3.50 ± 2.32	2.75 ± 1.16	3.90 ± 1.86***	1.00 ± 0.89***	2.67 ± 1.75
**BuC**	40	68	6	1.70	1.35 ± 1.69	2.05 ± 1.76	1.20 ± 1.40	1.30 ± 1.42	2.20 ± 2.15	2.10 ± 1.91	1.75 ± 1.75	2.15 ± 1.98	1.00 ± 1.10	0.83 ± 0.98
**CB**	40	88	5	2.20	2.25 ± 1.16	2.15 ± 0.99	1.80 ± 0.63	2.30 ± 0.95	2.30 ± 1.42	2.40 ± 1.17	2.00 ± 0.76	2.35 ± 1.27	1.83 ± 0.41	2.33 ± 1.21
**EG**	38	161	9	4.24	4.30 ± 1.03	4.17 ± 1.34	4.50 ± 1.58	4.40 ± 0.70	4.33 ± 1.00	3.67 ± 1.22	4.88 ± 0.64	4.00 ± 1.14	4.33 ± 1.03	4.00 ± 1.79
**GP**	36	67	6	1.86	1.81 ± 1.52	1.90 ± 1.21	1.33 ± 1.41*^c^	1.20 ± 1.03*^c^	3.25 ± 0.71*^a,b^	1.89 ± 1.17	1.86 ± 1.35	2.53 ± 1.18*^c,d^	0.83 ± 0.98*^b^	1.00 ± 1.10*^b^
**KS**	32	98	7	3.06	2.82 ± 1.33	3.33 ± 1.40	2.56 ± 1.24	2.86 ± 1.46	3.86 ± 1.07	3.11 ± 1.54	2.83 ± 1.33	3.44 ± 1.36	2.60 ± 1.34	2.60 ± 1.52
**LG**	40	145	8	3.63	3.85 ± 1.18	3.40 ± 1.23	3.10 ± 1.20	3.70 ± 1.06	3.80 ± 0.92	3.90 ± 1.60	3.63 ± 1.06	3.85 ± 1.27*	2.33 ± 0.82*	4.17 ± 0.75
**MO**	39	103	5	2.64	2.58 ± 0.96	2.70 ± 1.03	2.80 ± 0.79	2.00 ± 0.94	3.00 ± 1.05	2.78 ± 0.97	2.38 ± 0.74	2.89 ± 0.99	2.50 ± 1.05	2.33 ± 1.21
**PS**	39	151	9	3.87	3.45 ± 1.57	4.32 ± 1.42	2.90 ± 1.66	3.70 ± 1.64	4.60 ± 0.97	4.33 ± 1.41	3.63 ± 1.92	4.47 ± 1.17	3.17 ± 1.47	3.00 ± 1.67
**PL**	40	217	9	5.43	5.90 ± 1.29*	4.95 ± 1.15*	4.80 ± 1.03	5.60 ± 1.35	5.80 ± 1.40	5.50 ± 1.35	6.00 ± 1.51	5.65 ± 1.35	4.33 ± 0.82	5.00 ± 0.00
**SP**	40	162	9	4.05	3.90 ± 1.48	4.20 ± 1.44	3.40 ± 1.51	3.80 ± 1.48	4.20 ± 1.14	4.80 ± 1.48	3.88 ± 1.81	4.50 ± 1.32	2.67 ± 0.52	4.17 ± 1.33
**SM**	40	231	9	5.78	5.80 ± 1.70	5.75 ± 1.37	4.50 ± 1.58*^c,^**^d^	5.40 ± 1.17	6.40 ± 1.07*^a^	6.80 ± 1.23**^a^	5.25 ± 1.16	6.60 ± 1.14**	4.00 ± 1.90**	5.50 ± 0.84
**SA**	37	100	9	2.70	3.06 ± 1.66	2.37 ± 1.64	2.57 ± 1.72	1.70 ± 0.48	3.20 ± 1.69	3.33 ± 2.06	2.00 ± 1.10	3.25 ± 1.83	2.00 ± 1.22	2.17 ± 1.47

Cultural Importance (CI; arcsine square-root transformed) of 14 selected local woody species in dependence on the participants’ socio-economic background (sex, age, migration). Sex: female (n = 20), male (n = 20). Age: <20 years (n = 10), 20–39 years (n = 10), 40–59 years (n = 10), ≥60 years (n = 10). Migration: no_young (no migration < 40 years, n = 8), no_old (no migration ≥ 40 years, n = 20), temp (temporary migration, n = 6), perm (permanent migration, n = 6). Number of participants (# part). Number of citations (# cit). Number of use-categories (# cat). Data shown: mean values ± standard deviation. Significant differences between groups (a-d) determined by Tukey post-hoc tests. Levels of significance: *, *p* < 0.05; **, *p* < 0.01; ***, *p* < 0.001.

**Table 4:** Effects of socio-economic variables on uses of woody species

**Fixed effects**	**BD**	**BeC**	**BuC**	**CB**	**EG**	**GP**	**KS**	**LG**	**MO**	**PS**	**PL**	**SP**	**SM**	**SA**
**Intercept**	1.614	1.322	0.588	0.876	1.594	0.847	1.119	1.335	1.055	1.405	2.001	1.476	1.527	1.122
**age [a]**	ns	ns	ns	ns	ns	ns	ns	ns	ns	ns	ns	ns	0.009***	ns
**sex(men)**	ns	ns	ns	ns	ns	ns	ns	ns	ns	ns	ns	ns	ns	−0.052
**migration(yes)**	ns	−0.716**	ns	ns	ns	−0.934**	ns	ns	ns	ns	−0.296*	ns	ns	0.200
**age:sex(men)**	ns	ns	ns	ns	ns	ns	ns	ns	ns	ns	ns	ns	ns	ns
**sex(men):migration(yes)**	ns	ns	ns	ns	ns	ns	ns	ns	ns	ns	ns	ns	ns	−0.913*
**age:migration(yes)**	ns	ns	ns	ns	ns	ns	ns	ns	ns	ns	ns	ns	ns	ns
**age:sex(men):migration(yes)**	ns	ns	ns	ns	ns	ns	ns	ns	ns	ns	ns	ns	ns	ns

Parameter estimates for the effects of the socio-economic variables of age (continuous), sex, migration, and all possible two-way-interactions on the number of use-types mentioned for 14 selected species (see Table 1). Results based on the minimal adequate generalised linear models (GLM) with count data as response variables. Significance of main effects determined by F-tests (for quasi-Poisson error distribution) and likelihood ratio tests (for Poisson error distribution). Terms: ns, not significant; levels of significance: *, *p* < 0.05; **, *p* < 0.01; ***, *p* < 0.001.

**Table 5:** Effects of socio-economic variables on uses within use-categories

**Fixed effects**	**con**	**env**	**fie**	**fod**	**food**	**fuel**	**med**	**oth**	**tool**
**Intercept**	−0.994	−0.714	−2.356	−0.355	−2.881	−0.765	−1.853	−2.651	−2.537
**age [a]**	ns	ns	ns	ns	ns	ns	0.013**	0.014*	0.011**
**sex(men)**	ns	ns	ns	ns	ns	ns	ns	ns	ns
**migration(yes)**	−0.391**	ns	ns	−0.377**	ns	ns	ns	−0.975**	ns
**age:sex(men)**	ns	ns	ns	ns	ns	ns	ns	ns	ns
**sex(men):migration(yes)**	ns	ns	ns	ns	ns	ns	ns	ns	ns
**age:migration(yes)**	ns	ns	ns	ns	ns	ns	ns	ns	ns
**age:sex(men):migration(yes)**	ns	ns	ns	ns	ns	ns	ns	ns	ns

Parameter estimates for the effects of the socio-economic variables of age (continuous), sex, migration, and all possible two-way-interactions on the number of use-types mentioned for 14 selected species (see Table 1) within nine use-categories: construction (con), environmental use (env), field use (fie), fodder (fod), food, fuel, medicine (med), other use (oth), tool. Results based on the minimal adequate generalised linear mixed models (GLMM) with count data as response variables, and species and participants as crossed random effects. Significance of main effects determined by likelihood ratio tests. Terms: ns, not significant; levels of significance: *, p<0.05; **, p<0.01.

## Results

**Figure 5:**
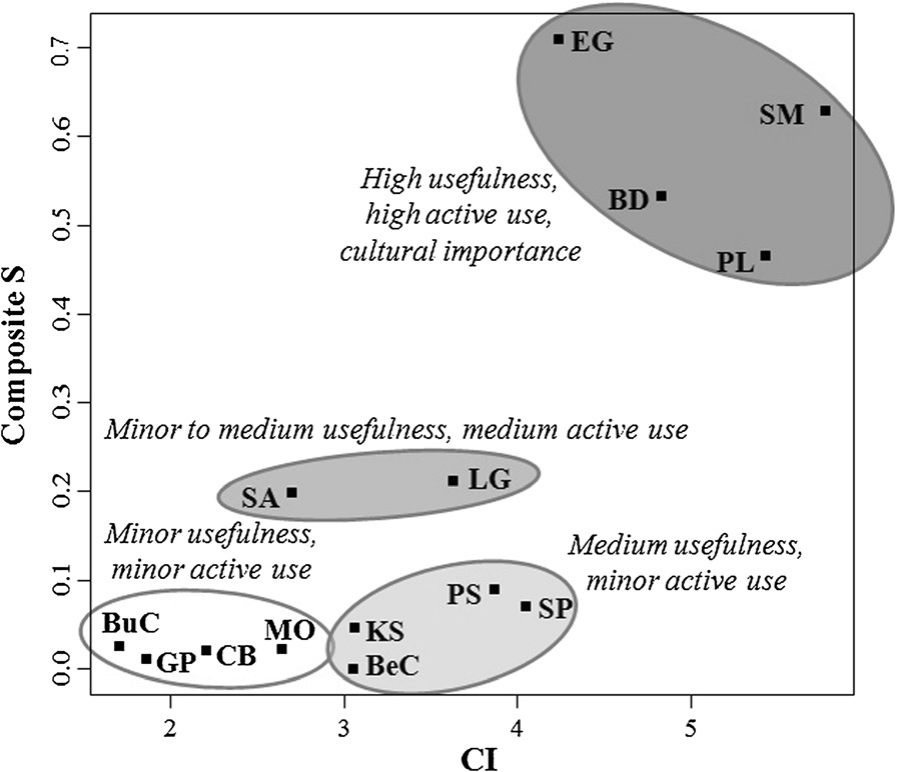
**Cultural Importance and Composite Salience of woody species.** Cultural Importance (CI) in relation to Composite Salience (Composite S) of 14 selected woody species: *Baccharis dracunculifolia* (BD)*, Berberis commutata* (BeC), *Buddleja coriacea* (BuC), *Clinopodium bolivianum* (CB), *Eucalyptus globulus* (EG), *Gynoxys psilophylla* (GP), *Kaunia saltensis* (KS), *Lepechinia graveolens* (LG), *Minthostachys ovata* (MO), *Polylepis subtusalbida* (PS), *Prosopis laevigata* (PL), *Sambucus peruviana* (SP), *Schinus molle* (SM), *Senna aymara* (SA).

During the freelisting exercises, the participants mentioned a total of 33 woody plants (n = 207 plant-reports), 64% of which were naturally-growing native species, 15% cultivated native species (e.g. *Buddleja coriacea,* kishuara; not naturally growing in the study area but originating from Bolivia), and 21% cultivated exotic species. Trees (46%) were the most frequently-mentioned woody life-form. All of the 14 selected species considered promising for use in agroforestry (Table 1) were also mentioned by the participants during the freelisting exercises, except for *Berberis commutata* (churisik’e). Calculation of the selected species’ CI values was based on a total of 1906 use-reports grouped into nine use-categories, the most cited of which were fodder (17.8% of use-reports), fuel (17.3%), and environmental uses (15.1%). Among the 14 selected species, *S. molle* (molle), *Prosopis laevigata* (thaqo, algarrobo), *Eucalyptus globulus* (eucalipto, kalisto), and *B. dracunculifolia* (t’ola) were assessed as the culturally most important species exhibiting the highest Composite S and CI values (Figure 5). Between both indices, a highly significant and strong correlation (R = 0.811, *p* < 0.001) was found.

The evaluation of Composite S showed that *Lepechinia graveolens* (raqacho, raga raga) was more important for men than for women (F_(1,38)_ = 5.424, *p <* 0.05), and less important for participants under 20 than those aged 20 to 39 years (F_(3,36)_ = 3.198, *p <* 0.05). Further significant age-effects on the intracultural variation in Composite S were detected for *Polylepis subtusalbida* (kewiña, queñua) (F_(3,36)_ = 3.238, *p <* 0.05) and *P. laevigata* (F_(3,36)_ = 4.717, *p <* 0.01). With regard to migration, significant intracultural variation was found for *E. globulus* (F_(3,36)_ = 4.660, *p <* 0.01), *P. laevigata* (F_(3,36)_ = 3.723, *p <* 0.05), and *Sambucus peruviana* (sauco) (F_(3,36)_ = 3.307, *p <* 0.05) (Table 2).

Significant intracultural variation in CI was found for *P. laevigata,* which could therefore suggest that it was culturally more important to women than men (F_(1,38)_ = 6.209, *p <* 0.05). *B. commutata* (F_(3,36)_ = 3.801, *p <* 0.05), *Gynoxys psilophylla* (k’apa towi, loma towi, k’apa k’apa) (F_(3,32)_ = 5.001, *p <* 0.01) and *S. molle* (F_(3,36)_ = 6.197, *p <* 0.01) were assumed to be culturally more important to elder than younger participants. Intracultural variation according to migration was found for *B. commutata* (F_(3,36)_ = 6.065, *p <* 0.01), *G. psilophylla* (F_(3,32)_ = 4.758, *p <* 0.01), *L. graveolens* (F_(3,36)_ = 3.599, *p <* 0.05) and *S. molle* (F_(3,36)_ = 6.587, *p <* 0.01), which were therefore suggested to be culturally more important to elder permanent residents than temporary migrants, and also to permanent migrants in the case of *G. psilophylla* (Table 3). A test of whether the significant intracultural variation in CI was based on specific use-categories showed that tool uses of *B. commutata* (<20 lower than 40–59 years; χ^2^_(1)_ = 7.273, *p* < 0.01), fodder uses of *G. psilophylla* (20–39 lower than 40–59 years; χ^2^_(1)_ = 8.128, *p* < 0.01) and food uses of *S. molle* (<20 lower than 40–59; χ^2^_(1)_ = 4.267, *p* < 0.05; <20 lower than ≥60 years, χ^2^_(1)_ = 10.208, *p* < 0.01) were mentioned significantly more frequently by elder than by younger participants. Furthermore, elder permanent residents emphasised fodder (χ^2^_(1)_ = 5.417, *p* < 0.05) and tool uses (χ^2^_(1)_ = 5.621, *p* < 0.05) of *B. commutata* and environmental uses of *S. molle* (χ^2^_(1)_ = 4.139, *p* < 0.05) more than temporary migrants.

In terms of total numbers of use-types known, we registered 2099 reports of 85 use-types across nine use-categories (n = 14 species). We found no significant effects of sex on the number of use-types known of the 14 selected plants. In contrast, knowledge of use-types increased significantly with age in case of *S. molle* (χ^2^_(1)_ = 11.107, *p* < 0.001), and was significantly lower for migrants than permanent residents for *B. commutata* (F_(1,38)_ = 8.787, *p* < 0.01), *G. psilophylla* (χ^2^_(1)_ = 9.746, *p* < 0.01), and *P. laevigata* (χ^2^_(1)_ = 4.596, *p* < 0.05). Furthermore, female migrants knew significantly more use-types of *Senna aymara* (motocho, motochila) than male migrants (χ^2^_(1)_ = 3.887, *p* < 0.05) (Table 4). Regarding the effects of sex, age, and migration on the number of use-types mentioned within the different use-categories, knowledge increased significantly with age concerning medicine (χ^2^_(1)_ = 9.347, *p* < 0.01), tools (χ^2^_(1)_ = 7.577, *p* < 0.01), and other uses (χ^2^_(1)_ = 5.881, *p* < 0.05). In addition, it was significantly lower for migrants than permanent residents regarding construction (χ^2^_(1)_ = 7.239, *p* < 0.01), fodder (χ^2^_(1)_ = 9.534, *p* < 0.01) and other uses (χ^2^_(1)_ = 7.335, *p* < 0.01) (Table 5).

## Discussion

### Culturally important agroforestry species

**Figure 6:**
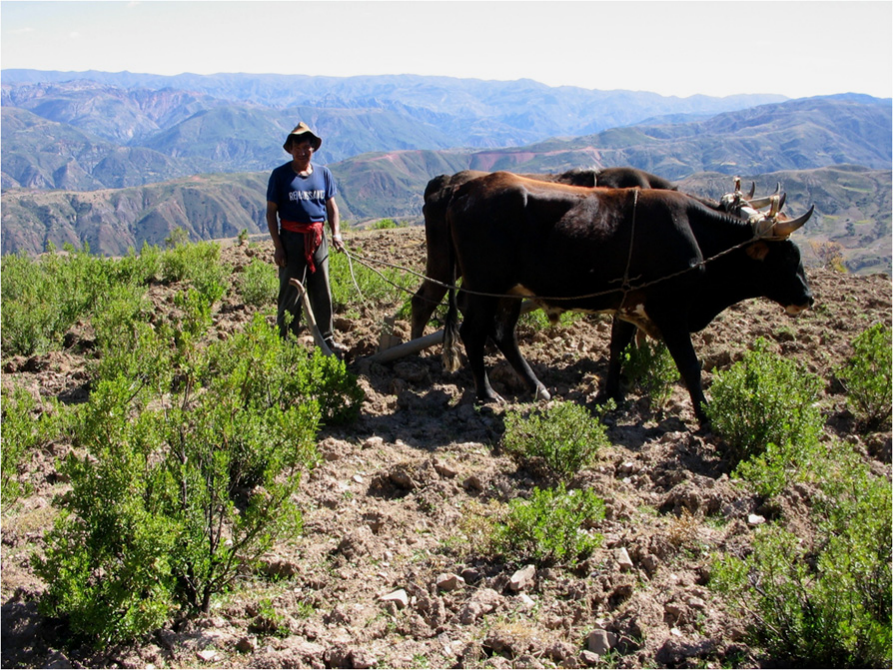
**Importance of *B. dracunculifolia *for the restoration of soil fertility in fallow land.** Photograph by R. Brandt.

Local people tended to give higher cultural importance to trees (e.g. *S. molle*) than shrubs (e.g. *L. graveolens*), as demonstrated by both ethnobotanical indices used (Composite S, CI) (Figure 5). This is supported by another study carried out in the same area [4] that revealed that sociocultural plant values increased with plant height and timber availability. This may be explained by the scarcity of timber needed for fuelwood and construction in the region. However, some frequently-growing shrubs such as *B. dracunculifolia* were also intensively used as fuelwood, for livestock fences, and in restoration of soil fertility in fallow land (Figure 6), and were thus highly valued by local people. This suggests that cultural importance and use-values of woody plants may not depend on their life-form only, but rather on their availability and accessibility (see also [4,8,9,46], especially in the case of frequently-used fuel plants [47]. Furthermore, plants’ specific attributes (e.g. hard wood, tasty fruits) that exclusively meet important subsistence needs increase their cultural importance [10]. *S. molle*, *P. laevigata*, *E. globulus*, and *B. dracunculifolia* were the culturally most important species in the study area (Figure 5) due to their high socio-economic and cultural values and high ecological apparency. However, in contrast to the native species mentioned, *E. globulus* was no promising agroforestry species [4] due to its potential negative effects on cultivated crops [48] and the environment (soil, water, biodiversity) [49,50]. Apart from cultural importance, the plants’ ecological values must therefore also be considered in the assessment of suitable woody species for agroforestry [4].

### Sex-specific valuation and knowledge of woody plants

Sex plays only a minor role in the intracultural variation of knowledge of the selected 14 woody plants in the study area, as no significant differences were found between women and men. The hypothesis about sex-specific differences in ethnobotanical knowledge and valuation in accordance with existing gender roles was not confirmed. This contradicts other studies [12,16,51] that showed sex-differentiated knowledge at the level of specific use-categories, which were explained by labour distribution between sexes, with men being experts in construction material and women knowing more about medicinal plants. However, the results are only partially comparable, because our research refers to selected woody species with potential for use in agroforestry while the studies mentioned also considered other life-forms (e.g. herbs) and plant uses (e.g. medicine). Furthermore, slightly significant sex-specific differences in cultural importance (Composite S) were shown in our study, but only for *L. graveolens*, which was more important for men than for women. This may be due to the shrub’s importance as a “green fertiliser” for soil fertility restoration on agricultural plots during fallow periods. Observing these plots and ploughing fallow fields is a typical men’s labour domain. Additionally, men were more involved in soil conservation project activities (previously described), which may have increased their awareness of the environmental services provided by plants. In contrast, *P. laevigata* was suggested to be culturally slightly more important for women than for men. This was surprising, because the species is commonly used for creating tools, which is typical men’s labour [33]. However, it also provides other uses (e.g. food, fodder), which is possibly the reason for the result. Indeed, these are specific examples of intracultural variation in knowledge and valuation of plants that are mainly used as organic fertilisers and tools and thus, may not represent general patterns. However, seen as preliminary results and observations they may stimulate further exploration in future studies. The overall negligible effect of sex on the intracultural variation in plant knowledge and valuation of the selected woody species found in our study is consistent with Lozada et al. [22] who explained this finding by their observation that both sexes may experience the selected plants similarly, despite their different social roles. Another explanation is that the highly developed horizontal knowledge transmission among neighbours and peers from the same generation reported in our study area [24] might have resulted in men and women sharing knowledge of medicinal and other active plant uses. This is especially true in view of increasing temporary migratory activities and specific familial circumstances (e.g. diseases or death of family members), which tended to lead to less fixed and more complex and dynamic gender roles in the region [33].

### Generational differences in the valuation and knowledge of woody plants

Significant differences according to age were detected in the cultural importance of several woody species with potential agroforestry use. This confirms our hypothesis of a decrease in plant knowledge and valuation among the youth, regarding, for instance, *P. subtusalbida* and *B. commutata*. Both tree species are characteristic of the potential vegetation of endangered high-Andean *Polylepis* forests [52], which are widely suppressed through human land and resource use [53,54]. The *Polylepis* forests growing in the area, for instance, have been drastically reduced since the Agrarian Reform of 1957 through the extension of agricultural plots, grazing, and timber use [55]. The fact that the cultural importance of *P. subtusalbida* and *B. commutata* was lower for younger than for elder peasants may illustrate that decreasing abundance results in decreasing use (according to the “ecological apparency hypothesis”, see [8]), and in decreasing valuation [56]. Strikingly, the two ethnobotanical indices applied indicated a different relative cultural importance for both species. Composite S values, based on freelisting, were relatively low and indicated minor active use. In contrast, relatively high CI values resulted from the semi-structured interviews and increased with age, possibly indicating an important passive knowledge. Thus, low active use of these two species may not be equal to low usefulness, suggesting that the use knowledge of these plants was mainly passive and more likely a relic from the past. This may be attributed to decreasing availability and substitution by other species (e.g. *E. globulus*) for fuel and construction use, as also observed in other ethnobotanical studies, e.g. [56]. Besides, obsolete use-types (e.g. dye, coal), as reported by Brandt et al. [4], can explain the decreasing cultural importance among the younger generation. We may consequently postulate that the ethnobotanical knowledge of the rare and seldom-used species *P. subtusalbida* and *B. commutata* is vulnerable to being lost. Even if knowledge is passive and no longer practiced, it may reflect the traditional sociocultural role of agroforestry species. In the interviews, the answers regarding *P. subtusalbida* and *B. commutata* showed abundant evidence for the paramount importance of such forests as a part of local people’s collective memory and identity. The drastic reduction of native species, in turn, was interpreted by Boillat et al. [57] as an effect of the increasingly disrespectful attitude towards the “natural expressions of Mother Earth”. Furthermore, these results illustrate that different methods combined for data collection (freelisting, semi-structured interviews) and analysis (Composite S, CI) may allow for more precise estimates on whether people have a passive or active ethnobotanical knowledge (Figure 5), which in turn provides a broader understanding of cultural plant importance [14]. While a certain overlap between active (Composite S) and passive knowledge (CI) is inevitable, it is interesting to note a marked difference in the ranking positions of species in both indices, such as in the cases of *P. subtusalbida* and *B. commutata.* However, further studies on the use of such method combinations for the evaluation of passive and active plant use knowledge should be conducted in order to refine them and confirm their relevance. This could be done, for instance, also by combining interviews on plant use knowledge with observations and freelistings on actual plant use.

Native *S. molle* was not only less culturally important for the youth, but it was also the only species that was significantly less known by young participants compared to elders. Unlike the cases of *P. subtusalbida* and *B. commutata*, this cannot be explained by the species’ decreasing ecological apparency. In fact, an upslope migration of *S. molle* (up to 3500 m.a.s.l.) has been locally observed, resulting from practices of natural regeneration management [4], and possibly also supported by increasing temperatures due to climate change [58]. The lower knowledge of young peasants may rather reflect a decreasing application of traditional plant uses of *S. molle* (e.g. “chicha de molle” - alcoholic beverage, medicinal uses) than by elders. This is in line with the significant generational difference in medicinal knowledge shown by our study. A positive correlation of medicinal knowledge with age has frequently been observed [12,16,17,23,38]. This difference may simply reflect the fact that learning about medicinal plant uses is a life-long process, and is important during adulthood [8,24]. Another explanation is that elder people are more likely to be ill and in closer contact with medicine [17]. However, declining knowledge among the youth has also been interpreted as an indicator of medicinal knowledge loss [38]. As many young people migrate, they take less part in collaborative familial activities of medicinal plant use and experimentation and thus, may have fewer or no opportunities to acquire new knowledge or reactivate passive knowledge through instructions or by means of observation and imitation [22]. However, a previous study in the same area showed that there was no loss of knowledge of local natural remedies (including plants, animals, minerals) from one generation to the next, but rather a transformation of knowledge on specific medicinal uses (e.g. respiratory diseases), which could simply reflect differences in health needs among different generations [25]. In the present study, the decreasing medicinal knowledge of woody species observed among the young generation possibly indicates an overall transformation of woody plant use. Our results indeed show that timber uses are increasing in importance compared to the uses of other plant parts, especially for the application as fuelwood or livestock fences, which might be associated with the scarcity of timber in the region [4].

### Effects of age and migration on the valuation and knowledge of woody plants

A combined effect of age and migration on the cultural importance of woody plants was shown for several species (e.g. *B. commutata*, *G. psilophylla*, *S. molle*); these species were of highest importance for elder permanent residents and less important for young migrants. However, we found no significant differences in cultural importance between young migrants and non-migrants, meaning that the observed differences were due to age rather than migration. Thus, this result did not confirm our hypothesis that migration led to a decrease in plant knowledge and valuation. A combined effect of age and migration was also found for the cultural importance of *P. laevigata*; it was significantly less known by migrants and had lower Composite S for younger compared to elder people. As this species is mostly used for making agricultural tools, this in turn corresponded with increasing knowledge of tool uses with age. On the one hand, decreasing availability and declining quality (length, thickness, and hardness of branches) of plants for crafting tools were often reported by the participants. According to the ecological apparency hypothesis, this possibly led to less knowledge of tool uses among the youth. On the other hand, our results are in line with young peasants’ decreasing commitment to traditional farming that is increasingly supplemented by off-farm activities for insuring and diversifying their income in the face of production risks [59].

The exotic *E. globulus* was especially important for young permanent residents and temporary migrants. This is in accordance with the general trend of *Eucalyptus* plantations as key components of rural livelihood strategies in the Central Andes [60], because the species is not only used in subsistence farming, but can be commercialised due to its dominance in Andean timber markets [31]. Marketability was also shown in other ethnobotanical studies to be a crucial factor in successfully integrating trees into agroforestry systems, e.g. [61]. In our study area, *Eucalyptus* plantations can therefore be considered a local strategy to raise and diversify income and mitigate agro-pastoral production risks, even though this exotic species implies, for instance, potential allelopathic effects on cultivated crops [48] and natural vegetation [49,50]. *Eucalyptus* is therefore not recommended for use in agroforestry systems [4]. Permanent migrants in contrast may give less importance to this species as they have other income sources (e.g. off-farm labour, crops such as coca cultivated in the lowlands).

The knowledge of other use-categories (e.g. domestic plant uses) was significantly affected by migratory activities, which confirmed our hypothesis of a decrease in plant knowledge and valuation according to this factor. For example, migrants mostly substituted wooden kitchenware by easily purchasable material (e.g. plastic pots), and were less aware of using native trees (e.g. *P. subtusalbida*) in construction, opting instead to use the timber of exotic trees (*E. globulus, Pinus* spp.). As illustrated by these results, migration requires progressive adaptation to changing socio-economic, cultural, and environmental surroundings [18]. This involves the assimilation and use of new plants and practices as well as adaptation or abandonment of traditional plants and uses [19,20].

## Conclusions

We believe that our investigation of the intracultural variation in the valuation and knowledge of plants contributes to a better understanding of the societal dynamics that underlie the attitudes and practices of indigenous Andean peasants towards woody plants. This in turn may help external actors support community-based agroforestry initiatives that are adapted to the dynamic socioecological context of the land users. The fact that the results do not reveal strong general patterns – but, rather, species-specific effects of sex, age, and migration – may reflect the level of precision of traditional knowledge and valuation at our study site: people seem to focus on specific species rather than on the woody vegetation in general. In this regard, the combination of two indices (Composite S and CI) may be of particular value in differentiating between active and passive ethnobotanical knowledge of woody plants and the type of transformation that these knowledge categories are undergoing.

In our study area, the valuation and knowledge of woody species and their uses showed a significant decline in younger and migrating peasants, but sex played a negligible role overall. Thus, when establishing community-based agroforestry systems, we recommend paying particular attention to the selection of species that meet the needs and interests of the young (temporarily) migrating peasant generation. In this regard, as the example of *Eucalyptus* illustrated, the importance of marketable species should be recognised, even though this specific exotic species is not recommended for use in agroforestry for ecological reasons. We thus suggest selecting ecologically sound species, which provide subsistence and potential commercial uses. Positive examples are, for instance, the native trees *S. molle* with its aromatic, antimicrobial, and insecticidal properties [62,63], and *P. laevigata,* which provides highly nutritive pod flour [64]. Furthermore, as the case of native *Polylepis* forest species (*P. subtusalbida*, *B. commutata*) showed, plants which are no longer actively used may still be of high sociocultural importance for collective identity. By privileging native trees and shrubs with important cultural value, we believe that agroforestry initiatives – in addition to offering socio-economic and environmental services – can play important roles in recovering elements of the lost ancient landscape that still forms part of local people’s collective identity.

## Competing interests

The authors declare that they have no competing interests.

## Authors’ contributions

RB designed the study, collected and analysed the data, and drafted the manuscript. SLMS, SR and IH helped with the study design and data interpretation. SL participated in the data analysis. All authors contributed to the revisions of the manuscript, and approved its final version. The photographs were taken by RB and SLMS.
